# Pre-Existing Disability and Its Risk of Fragility Hip Fracture in Older Adults

**DOI:** 10.3390/ijerph16071237

**Published:** 2019-04-07

**Authors:** Jayeun Kim, Soong-Nang Jang, Jae-Young Lim

**Affiliations:** 1 Institute of Health and Environment, Seoul National University, Seoul 08826, Korea; kimjayeun@gmail.com; 2 Red Cross College of Nursing, Chung-Ang University, Seoul 06974, Korea; 3 Department of Social and Behavioral Sciences, Harvard T.H. CHAN School of Public Health, Boston, MA 02115, USA; 4 Department of Rehabilitation Medicine, Seoul National University Bundang Hospital, Seoul National University Institute on Aging, Seoul 13620, Korea; drlim1@snu.ac.kr

**Keywords:** disability, hip fracture, gender, severity, comorbidity

## Abstract

*Background*: Hip fracture is one of the significant public concerns in terms of long-term care in aging society. We aimed to investigate the risk for the incidence of hip fracture focusing on disability among older adults. *Methods*: This was a population-based retrospective cohort study, focusing on adults aged 65 years or over who were included in the Korean National Health Insurance Service–National Sample from 2004 to 2013 (*N* = 90,802). Hazard ratios with 95% confidence interval (CIs) were calculated using the Cox proportional hazards model according to disability adjusted for age, household income, underlying chronic diseases, and comorbidity index. *Results*: The incidence of hip fracture was higher among older adults with brain disability (6.3%) and mental disability (7.5%) than among those with other types of disability, as observed during the follow-up period. Risk of hip fracture was higher among those who were mildly to severely disabled (hazard ratio for severe disability = 1.59; 95% CI, 1.33–1.89; mild = 1.68; 95% CI, 1.49–1.88) compared to those who were not disabled. Older men with mental disabilities experienced an incidence of hip fracture that was almost five times higher (hazard ratio, 4.98; 95% CI, 1.86–13.31) versus those that were not disabled. *Conclusions*: Older adults with mental disabilities and brain disability should be closely monitored and assessed for risk of hip fracture.

## 1. Introduction

Individuals with disabilities are vulnerable to “secondary conditions”, where there is an ever-present risk of further disease or accident/injury, as either a direct or indirect result of the pre-existing physical, mental, or social disability. Prevention of such secondary conditions is of the utmost importance for promoting the health of individuals with disabilities. However, the association between disability and hip fracture has long been overlooked, as disability was typically regarded and treated as a complication ensuing from a hip fracture. Although increased hip fracture incidence was detected among individuals with functional disability [1,2,3], a more targeted approach, i.e., one that takes disability type and severity into consideration, is necessary to increase public awareness regarding prevention of hip fracture, and to develop a customized rehabilitation program. Individuals who experience hip fracture utilize more medical services and have higher hospital admission rates, thus incurring higher medical costs during the first year following osteoporotic hip fracture [4] compared to patients who have not experienced fractures. Post-fracture rehabilitation for patients with pre-existing disabilities is likely to be more complex and of longer duration than it is for patients without pre-existing conditions, which is also associated with considerable expense [5]. Furthermore, disability as a concept is broad and the disabled population is not homogeneous; thus, fracture risk among individuals with disabilities should be assessed in conjunction with other risk factors and demographic characteristics. For example, bone mineral density, which significantly affects bone fracture risk, varies according to race, gender, and area of residence [5]. Nutrition quality (including calcium intake), fertility, and physical activity also vary according to cultural, social, and economic circumstances.

As chronic illnesses become more widespread, as a result of aging populations and the greater likelihood of survival following physical injury, disability is becoming more prevalent. Hip fractures, in particular, are among the most serious concerns with respect to long-term healthcare in an aging society. As such, targeting groups at high risk of hip fracture is imperative for the development and delivery of robust and appropriate prevention and intervention strategies, and tailored patient education regarding risk avoidance and rehabilitation. Based on this background, we hypothesized that there is a difference of relative risk on hip fracture between pre-existing disability type and severity, because of the difference of underlying diseases and sociodemographic characteristics among older adults.

This study, therefore, aimed to assess the incidence of hip fracture, and to explore the association between hip fracture and various demographic characteristics, including the type and severity of pre-existing disability and the presence of an underlying condition, using medical insurance claim data in Korea. Nationwide insurance claim data from all hospitals were collected, and cohort data have been collated by the Korean government since 2002. We determined the hazard ratios for well-known risk factors, including age, socioeconomic status, disability, and underlying disease, and further estimated the effect of disability status, according to gender.

## 2. Materials and Methods

### 2.1. Study Design

The study design was observational study with retrospective cohort analysis using the national health insurance claims dataset.

### 2.2. Data Sources and Size

We used data from the National Health Insurance Service–National Sample Cohort (NHIS–NSC) for 2002–2013. NHIS–NSC is a population-based cohort established by the National Health Insurance Service (NHIS) in South Korea [6]. This cohort includes 1,025,340 people (as of 2002, approximately 2.2% of the entire Korean population) who were randomly selected to represent the general Korean population. The NHIS–NSC data included demographic information, such as gender, age, area of residence, household income, and type of health insurance. Inpatient and outpatient medical care utilization data, including primary and subsidiary reasons and dates of access, was also available.

### 2.3. Participants

Our study population was 90,802 persons of the NHIS–NSC aged 65 years and over; 790 individuals were excluded because they had experienced hip fracture prior to the follow-up start date of July 1, 2004. We established 2002 to 2004 as the run-in period, and excluded patients who had received a diagnosis, or had a history, of hip fracture during this time. Consequently, hip fracture events could potentially be followed up over almost 9.5 years, if not excluded from analysis due to death, emigration, or other reasons, prior to the study’s conclusion in 2013.

All reasons for accessing medical care, including hip fracture, were recorded using the International Classification of Diseases, 10th revision (ICD-10) [7]. We defined fragility hip fracture patients as those with: (1) a primary or subsidiary cause of medical service usage, classified as ‘S72.0 (Fracture of the femoral neck)’, ‘S72.1 (Pertrochanteric fracture)’, or ‘S72.2 (Subtrochanteric fracture of femur)’, and (2) no prior primary or subsidiary medical service usage, classified as ‘S72.0’, ‘S72.1’, or ‘S72.2’, prior to participation in the study. Using the cohort data, the incidence rate of hip fracture per 100,000 per decade was calculated as the crude incidence rate among the final study population of 90,012 and 2015 census data from the Statistics Korea was used for age- and gender-standardization.

### 2.4. Variables

Demographic characteristics and socioeconomic status, including gender, age, type of health security (i.e., health insurance vs. medical aid), household income, and disability were based on subjects’ 2004 health insurance qualification records. Korea implements a compulsory National Health Insurance system, and the Medical Aid program is the social security-covered medical cost for those deemed to be below the basic standard of living. Data regarding household income was classified into 10 groups; the total annual income of the head of household was also recorded.

The type and severity of disability are classified into eight categories based on the National Disability Registry system, including limb disability, brain disability, visual disability, auditory impairment, mental retardation, mental disease, renal impairment, and the others. The disability assessment for National Registry is made by the specialist doctors of the disability diagnosis in the professional institutions for each type of disability when the disabled status continues for one year or more. In this study, the severity of disabilities were classified as ‘severe’ (level 1–level 3 of National Registry) or ‘mild’ (level 4–level 6), on an individual basis according to National Registry criteria which is classified by medical perspective. We created three further categories: external disability (including limb disability, brain disability, visual disability, and auditory impairment), mental disability (including mental retardation and mental disease), and internal organ and other disability (such as renal impairment, etc.).

Subjects were categorized by baseline age (65−69, 70−74, 75−79, 80−84, or 85+ years) to compare the estimated age-specific effect. Household income was stratified into three groups according to income decile: low (0–2), middle (3–7), and high (8–10) deciles. Information regarding underlying medical conditions was collected on stroke (I60–I69, excluding G45.3, I67.0, I67.3, and I68.0), Parkinson’s disease (G20), and osteoporosis (M81). Additionally, the score on the Charlson Comorbidity Index (CCI) [8], a method of categorizing patient comorbidities based on the ICD diagnostic codes within administrative data (e.g., hospital records) and which has been widely used for risk adjustment in outcome studies [9,10], was determined for each subject using 2002–2003 medical care utilization records. We looked up their medical records for individual patients among the study populations between 2002 and 2003 and excerpted the diagnosis records and calculated individual CCI.

### 2.5. Statistical Methods

Descriptive statistics for the study population are presented as *N* (%) for dichotomous variables, and as means ± standard deviation for continuous variables. Fracture incidence rate, that is, the case number per 100,000 population during the study period between 2005 and 2013, was calculated as the age- and gender-standardized rate using 2015 population census data [11], as collected by Statistics Korea. We also present the rate of hip fracture during the study period, according to general characteristics, disability and chronic diseases.

We observed that 3943 people experienced hip fracture during the study period, and subsequently performed the Chi-Square test and Student’s *t*-test to compare general characteristics, including disability and underlying diseases, according to gender (men vs. women) and disability (disabled vs. non-disabled).

The Cox proportional hazards model [12] was used to investigate the association between disability type and severity of hip fracture. Follow-up concluded on the last date of medical care utilization for excluded subjects, and on the initial date of medical care utilization for hip fracture for case subjects. Age, gender, type of health security (i.e., health insurance vs. medical aid), household income, disability (including severity), CCI, and underlying diseases (stroke, Parkinson’s, and osteoporosis) were considered as potential risk factors. The analyses were repeated separately for men and women, and for disabled and non-disabled individuals. Additionally, we examined hazard ratios for each combination of disability type, severity and gender. Interaction effects with regard to these permutations were also investigated. All analyses were conducted using SAS software (ver. 9.4; SAS Institute Inc., Cary, NC, USA), and *p*  <  0.05 was considered statistically significant.

### 2.6. Ethics

This study was exempted from the requirement for ethical approval by the Institutional Review Board (IRB) of C University (#IRB No. 1041078-201607-HR-145-01). Informed consent was obtained when patient records and other data were collected from the hospitals. The data were subsequently anonymized and de-identified prior to analysis.

## 3. Results

### 3.1. General Characteristics of Study Participants

Baseline characteristics of the study population are presented in Table 1. There were 3943 cases (4.4%) of hip fracture among the total 90,012 participants. The majority of participants were females (60.6%) aged 65–74 (66.3%) years. The age- and gender-standardized hip fracture incidence rate was 4612 per 100,000 people over a 10-year period. In total, 9.0% had a disability of some description; 2.9% were severely disabled, and 2.9% had a mild disability. Limb disability was the most frequent type, followed by brain disability and auditory impairment. Osteoporosis was the most prevalent condition among the entire study population (12.9%).

Women were more likely to have experienced hip fracture (5.3%) than men (3.0%). As expected, older people with mild and severe disabilities had a higher incidence proportion of hip fracture compared to those without disabilities. Among the disability types, mental disability and brain disability were associated with higher proportion of hip fracture (7.5% and 6.3%, respectively). The mean follow-up time was 2861.4 ± 953.9 days.

### 3.2. General Characteristics of Patients with Hip Fracture

Table 2 presents the general characteristics of hip fracture patients during the study period, stratified according to gender and disability. Among these patients, women (*n* = 2877) greatly outnumbered men (*n* = 1066), and the age distribution differed according to gender. Disability and underlying disease prevalence, excluding Parkinson’s disease, differed according to gender, whereas type of health security and household income were similarly distributed across both genders.

Among the older adults who had experienced hip fracture, 11.4% had pre-existing disabilities (*n* = 451, 11.4%), and mild external physical disability was prevalent, at 7.7% (*n* = 304). Men with disabilities had proportionately greater experience of hip fracture (19.2%), compared to women (8.6%). Age, health security type, and CCI also exhibited different distributions between disabled and non-disabled older adults. Regarding the comorbidity conditions, increased risk of hip fracture was observed with stroke, Parkinsonism, and osteoporosis.

### 3.3. Hip Fractures: Disability Type And Severity, and Subgroup Analysis

Table 3 presents the risk factors for hip fracture, and gender- and disability-stratified hazard ratios. Hip fracture incidence rate ratio was 1.59 times greater among older adults with severe disabilities, and 1.68 times greater among those with mild disabilities. Overall, older age (years) was associated with increased hazard ratios and Medical Aid beneficiaries appeared to have lower risk of hip fracture. Regarding the comorbid conditions, hip fracture was observed more frequently among patients who had suffered strokes, Parkinson’s disease, and osteoporosis, compared to those with no history of these conditions and greater CCI was associated with higher hazard ratios (CCI = 0 vs. 1 ≤ CCI ≤ 2; CCI = 0 vs. CCI ≥ 3).

In the subgroup analyses according to gender, severe disability was more strongly associated with risk of hip fracture among men (hazard ratio, 2.02; 95% CI, 1.57–2.61) than among women (hazard ratio, 1.28; 95% CI, 1.01–1.64). There were also gender differences regarding the degree to which the risk of hip fracture increased with older age, with greater risk increases observed among women and smaller increases observed among men. Diagnoses of underlying chronic diseases were associated with increased hazard ratios for both men and women. 

In another Cox proportional hazards regression model, distinguished by disability in Table 3, women’s hazard ratio was also higher than that of men. The impact of age was lower among the disabled; for example, a hazard ratio of 1.57 was observed among individuals with disabilities aged 80–84 years, while a hazard ratio of 4.75 was observed among those without disabilities within the same age band.

**Figure 1:**
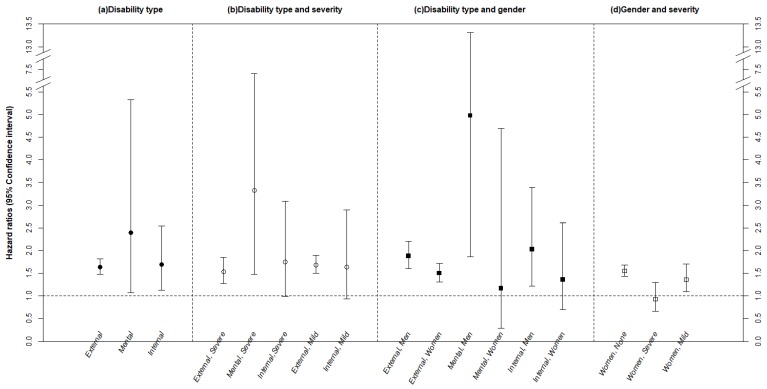
Hazard ratios (95% Confidence interval) for the hip fracture event according to the type of disability, severity and gender among older Korean. (**a**) Disability type, references=non-disabled in each type; (**b**) Disability type and severity, references = non-disabled; (**c**) Disability type and gender, references = non-disabled; (**d**) Gender and severity, references=men counterparts. CCI, Charlson comorbidity index. Note: The hazard ratios were estimated with adjustment for gender, age group, type of health security, household income, CCI and specified underlying diseases; gender interaction with mental disability was significant (gender × mental disability, *p* < 0.001 in (**c**).

We next investigated the risk associated with various combinations of disability type, severity and gender (Figure 1). We observed that all three disability types were associated with increased hip fracture incidence rate ratio, to a greater extent than was observed among the non-disabled study subjects (Figure 1a). External physical disability (including limb, brain, visual, and auditory impairment) was associated with higher hip fracture incidence rate ratio (hazard ratio, 1.64; 95% CI, 1.48–1.82), irrespective of disability severity or gender. However, the risk of hip fracture among people with mental disability varied according to gender and severity of disability. The overall risk of hip fracture among the mentally disabled, expressed as the hazard ratio, was 2.39 (95% CI, 1.07–5.33), but for severely mentally disabled patients, the hazard ratio was 3.32 (95% CI, 1.48–7.41). Hazard ratio increased for older men with mental disability (4.98; 95% CI, 1.86–13.31) compared to women, confirming a gender disparity, with a significant gender/mental disability interaction (*p* < 0.01, data not shown). Regarding internal organ impairment or disability, while the risk was elevated among disabled women compared to men with disabilities, there was no statistically significant gender interaction.

## 4. Discussion

In this longitudinal analysis, hip fracture incidence rate ratio was observed to occur over 1.6 times more frequently among older adults with pre-existing disabilities compared to older adults without disabilities. Incidence proportion of hip fracture were elevated among those with mental disabilities (7.5%) and brain disabilities (6.3%), versus those with other disability types. Mental disability was more strongly associated with elevated risk of hip fracture than were physical disabilities or afflictions of the internal organs. The highest risk group overall comprised men with mental disability, among all combinations of disability type, disability severity and gender.

Previous studies also concluded that higher fall incidence and accident rates among individuals with disabilities resulted in elevated incidence of hip fracture. For example, age-related differences in experience of hip fractures induced from the functionality, comorbidities, surgical features, baseline differences by pharmacologic treatments, complications and features at hospital discharge [1]. In addition, the conclusion of previous literature that the cognitive impairment levels were associated with the characteristics, comorbidities, pharmacology, and complications of older adults with hip fracture [2] also supports the importance of the prevention of secondary condition. A southern-European-based longitudinal study [13] suggested that self-reported disabilities, such as difficulty walking several blocks and climbing stairs, increased in the two years prior to hip fracture. However, few studies to date have distinguished the effects of disability type and severity, in addition to gender differences. The results of this study demonstrated that heterogeneity in disability should be recognized and considered, according to general characteristics, and disability type and severity.

Higher hip fracture risk was associated with mental disability in our study population. Hip fracture risk is likely to be associated with certain antipsychotic agents that are suspected of accelerating bone loss [14,15,16]. Risk factors for falls, fractures, lower bone mineral density and osteoporosis [17], including poor nutritional intake [18], are elevated among people with schizophrenia-spectrum disorders, according to findings that showed consistency across several meta-analyses investigating these disorders. Stubbs et al. (2014) suggested that people with schizophrenia-spectrum disorders were 2.5 times more likely to suffer from osteoporosis [19]. Another meta-analysis concluded that people with mental disorders were more likely to exhibit reduced bone mineral density in the femur and lumbar spine compared to matched controls without mental disorders [20]. Regarding the association between antipsychotic medication and risk of hip fracture, Wu et al.’s (2015) cohort study focused on antipsychotic medication, and demonstrated elevated hip fracture risk among schizophrenic subjects who had received such drugs [21]. Not all research findings have been consistent, however; for example, a study conducted to determine the association between anti-depressant medication and the incidence of falls among people with late-onset polio sequelae suggested that, while depression is related to the incidence of falls among polio survivors, fall incidence was not positively correlated with the use of anti-depressant or psychoactive medications [22]. Among the older adults, this might be not just related to Parkinson disease, but to all the neurological conditions, especially Alzheimer’s disease, that we can consider as an accelerated form of ageing including the Motor Risk Cognitive Syndrome [23,24]. Indeed, there are a number of studies that highlight the concept that ageing itself is a risk of fall and hip fracture also because of the gradual and physiological cholinergic depauperization (more accentuated in Alzheimer’s disease) [25,26]. When getting older we progressively lose the scheme of the gait and lower our center of gravity [27]. These are all considerations for the mechanism of the association between hip fracture risk among mentally disabled people. 

Nevertheless, the implementation of routine screening, as well as bone health promotion and intervention, is recommended to improve bone mineral density and thereby prevent fractures among older adults with mental disabilities. Considering the high incidence rate ratio of fracture among men with mental disabilities, specific attention should be paid to the prevention of fractures among this group. The provision of supportive care customized in terms of disability characteristics and gender is imperative for fracture prevention.

Generally, hip fracture is more prevalent among women than among men, and this also holds true among individuals with disabilities. In a 16-year longitudinal study conducted in Spain by the Zaragoza Dementia and Depression (ZARADEMP) Project, Lobo et al. (2017) reported that hip fractures were more frequent among women than among men (incidence rate ratio = 3.1), and that impaired ability to perform basic activities of daily living increased the risk among men (hazard ratio = 3.14); this study identified gender differences and disabilities preceding hip fracture among those aged 55+ years [13]. Kellie et al. (1990) investigated gender-, race-, and age-specific hip fracture rates based on Health Care Financing Administration data, and arrived at similar conclusions regarding white women compared to white men, and black women compared to black men [28]. Another study, conducted to estimate hip fracture incidence rates in the United States using non-federal hospital discharge data from the National Hospital Discharge Survey, reported that, following age-adjustment, the risk for white women was 2.7 times that for white men [29]. The reason for higher rates of hip fracture among women may be their lower body mass index (BMI) and lower bone mineral density, especially among older individuals [30].

Men with severe disabilities appear to suffer considerably more hip fractures than men without disabilities. In this study, the hazard ratio of severely disabled men was 2.02, while severely disabled women had a hazard ratio of 1.28 compared to women without disabilities. The impact of disability on hip fracture risk is thus higher among men. This may be partly attributable to the fact that men with disabilities continue to actively participate in more social, employment, and physical activities than women [31]; thus, disabled men have a greater exposure to risk factors for physical accidents or injuries than do disabled women. A possible alternative explanation concerns the fact that the disabilities were not self-reported; instead, individuals were officially registered as ‘disabled’ by the National Enforcement Rule of the Disabled Welfare Act. Since non-registered disabled people are mostly elderly (more than 60%) [31], and registered disabled people tend to be younger males, this may somewhat distort the picture regarding the impact of disability on fall and fracture risks.

Our findings indicate that the risk of hip fracture increases with age, a result that is consistent with those of previous studies [1,29,32] among both disabled and non-disabled people, though the gradient was steeper among non-disabled individuals. Owing to the strong association between disability rate and age, the association between age and hip fracture was attenuated among disabled people. We should take into account the frailty incidence according to age [33]. Hip fracture is one of the clinical features that are part of a more complex physical status in which the physical and cognitive status of the elderly is frequently associated with low physical activity, slow walking speed, self-reported exhaustion and weight loss, probably due to the association of diverse extrinsic conditions like inflammatory status and co-morbidities like hypertension, diabetes or heart disease, and also of intrinsic conditions like recognized genetic factors [34].

Underlying chronic diseases were also associated with increased hip fracture risk, which is consistent with the findings of several previous studies [35,36,37,38]. A systematic review and meta-analysis conducted by Luan et al. (2016) found that, independently, stroke more than doubled the risk of hip fracture (relative risk (RR) = 2.06; 95% CI, 1.68–2.52) [38]. Vestergaard et al. (2007) found that the association of Parkinson’s disease with any fracture had a crude odds ratio (OR) of 2.22 (95% CI, 1.99–2.46) in a case-control study [39]. The same study found that the association of Parkinson’s disease with hip fracture had an adjusted OR of 1.62 (95% CI, 1.24–2.13) among osteoporotic fractures [39]. Osteoporosis is a well-established risk factor for hip fracture, mostly as a result of a lowered level of bone mineral density [40]. In this study, we investigated comorbidities in terms of the CCI [9], and concluded that three or more comorbidities increased the risk of hip fracture among the general older population, but not among the disabled older population. This may be attributable to the homogeneity of morbidity among older disabled people, which obscures its influence on hip fractures.

The present study had several limitations. First, this study is designed based on retrospective cohort study and the data were gleaned from insurance claims, information concerning patients who did not attend hospital or claim premiums is absent. In addition, the residing conditions and environments of patients were not observable, and health behavior information was limited. Although extended to include as much as information using the data, it is still limited in including comprehensive risk factors related with fall and hip fractures. For example, the results seemed in line with recent literature that highlight cognitive dysfunction as a risk factor for fall and consequently hip fracture [2]. The mental disability in this study was vague and should need a deeper reflection in order to help medical professionals to better stratify the risk of hip fractures in the older adults. Second, the data regarding underlying conditions and medical care utilization for hip fracture were obtained in the form of ICD-10 codes; thus, if participants were not seen at a medical institution, the disease incidence or prevalence may have been under-estimated. However, the present study has important clinical implications because a real-world large sample sized data from a national database was used, and without selection bias.

Third, in the NHIS–NSC dataset, only the head of household’s income was considered when determining the monthly insurance fee deducted from the salary [41], and dependents do not always live with the head of household. Thus, the data that were used in this study may not accurately reflect the actual household incomes of the participants. The disability categories used the information in the registry system on this database, so comparison with the standard categories may be limited.

Finally, reliable clinical data regarding fragility fracture, treatment of fractures, bone density and the functional status measurements were absent in this study. We can perhaps assume that the over-reporting of geriatric hip fracture incidence may result from the inclusion of hip fractures caused by accidents. In addition, although we discussed the antipsychotic use or anti-osteoporosis medications as potentially being the explanation for increased or decreased fracture risk in patients with disability in the above description, the absent analysis of medication data is a limitation of the study. Thus, it should be discussed in a relevant analysis in further study. Environmental factors affecting hip fracture rates may include green spaces, according to their relationship with physical activity levels, and the maintenance of public sidewalks and other facilities [42]. However, the absence of such information as it pertains to the individuals in this study limited our ability to fully investigate the association between hip fracture risk and region of residence.

## 5. Conclusions

Although hip fracture risk was observed to generally increase among older adults with disabilities during the follow-up period, the hazard ratios for hip fracture differed according to disability type and severity, and also by gender. Our findings indicate the need for detailed identification of groups at risk of hip fracture. Intervention to mitigate the risk of hip fracture among the older disabled population should be tailored to take account of the heterogeneous characteristics of different disabilities. In particular, particular consideration should be given to older men with mental disabilities, to ensure implementation of appropriate strategies for prevention, intervention and promotion of patient education regarding rehabilitation.

## Figures and Tables

**Table 1:** General characteristics of the study participants in baseline National Health Insurance Service–National Sample Cohort (NHIS-NSC) cohort data.

Variables	Category	Total	Fracture Experience
		*N* (%)	*N* (%)
Total ^1^		90,012 (100.0)	3943 (4.4)
Hip fracture incidence rate ^2^ (per 100,000)		*—*	4612
Gender	Men	35,426 (39.4)	1066 (3.0)
	Women	54,586 (60.6)	2877 (5.3)
Age group	65–69	35,417 (39.3)	899 (2.5)
	70–74	24,348 (27.0)	970 (4.0)
	75–79	15,424 (17.1)	1004 (6.5)
	80–84	9183 (10.2)	702 (7.6)
	85+	5640 (6.3)	368 (6.5)
Type of health security	Health insurance	80,670 (89.6)	3639 (4.5)
	Medical aid	9342 (10.4)	304 (3.3)
Household Income ^1^	Low	23,624 (26.2)	970 (4.1)
	Middle	27,957 (31.1)	1206 (4.3)
	High	38,431 (42.7)	1767 (4.6)
Severity of disability	Non-disabled	81,945 (91.0)	3492 (4.3)
	Severe	2601 (2.9)	135 (5.2)
	Mild	5466 (6.1)	316 (5.8)
Type of disability			
External disability	Limb disability	3990 (4.4)	237 (5.9)
	Brain disability	1308 (1.5)	83 (6.3)
	Visual disability	981 (1.1)	47 (4.8)
	Auditory impairment	1157 (1.3)	54 (4.7)
Mental disability	Mental retardation	57 (0.1)	3 (5.3)
	Mental disease	40 (0.0)	3 (7.5)
Internal organ disability etc.	Renal impairment	161 (0.2)	8 (5.0)
	Other disability	373 (0.4)	16 (4.3)
Underlying diseases	Stroke	6604 (7.3)	371 (5.6)
	Parkinson	413 (0.5)	28 (6.8)
	Osteoporosis	11,622 (12.9)	713 (6.1)
Charlson comorbidity index	0	59,712 (66.3)	2522 (4.2)
	1–2	27,723 (30.8)	1293 (4.7)
	3+	2577 (2.9)	128 (5.0)
Follow-uptime (day), mean (SD, standard deviation)		2861.4 (953.9)	1939.0 (941.9)

^1^ Household income group were defined as three groups using income decile: Low (0–2), Middle (3–7), High (8–10). ^2^ Age and gender standardized incidence rate of hip fracture using 2015 population census.

**Table 2:** General characteristics of older adults who experienced hip fracture.

Variables	Category	Overall, *N* (%)	Gender, *N* (%)			Disability, *N* (%)		
			Men	Women	*X*^2^ or *t*-test (*p*-Value)	Disabled	Non-Disabled	*X*^2^ or *t*-test (*p*-Value)
Total ^1^		3943 (100.0)	1066 (27.0)	2877 (73.0)		451 (11.4)	3492 (88.6)	*X*^2^ = 3943.00 (<0.0001)
Gender	Men	1066 (27.0)	–	–		205 (19.2)	861 (80.8)	*X*^2^ = 87.59 (<0.0001)
	Women	2877 (73.0)	–	–		246 (8.6)	2631 (91.4)	
Age group	65–69	899 (22.8)	346 (38.5)	553 (61.5)	*X*^2^ = 78.13 (<0.0001)	179 (19.9)	720 (80.1)	*X*^2^ = 115.14(<0.0001)
	70–74	970 (24.6)	275 (28.4)	695 (71.6)		125 (12.9)	845 (87.1)	
	75–79	1004 (25.5)	225 (22.4)	779 (77.6)		94 (9.4)	910 (90.6)	
	80–84	702 (17.8)	144 (20.5)	558 (79.5)		39 (5.6)	663 (94.4)	
	85+	368 (9.3)	76 (20.7)	292 (79.3)		14 (3.8)	354 (96.2)	
Type of health security	Health insurance	3639 (92.3)	995 (27.3)	2644 (72.7)	*X*^2^ = 2.26 (0.1326)	397 (10.9)	3242 (89.1)	*X*^2^ = 13.00 (0.0003)
	Medical aid	304 (7.7)	71 (23.4)	233 (76.6)		54 (17.8)	250 (82.2)	
Household income ^2^	Low	970 (24.6)	248 (25.6)	722 (74.4)	*X*^2^ = 2.83 (0.2432)	126 (13.0)	844 (87.0)	*X*^2^ = 3.10 (0.2118)
	Middle	1206 (30.6)	346 (28.7)	860 (71.3)		130 (10.8)	1076 (89.2)	
	High	1767 (44.8)	472 (26.7)	1295 (73.3)		195 (11.0)	1572 (89.0)	
Type of disability ^1^	External physical disability	421 (10.7)	186 (44.2)	235 (55.8)	–	–	–	–
	Mental disability	6 (0.2)	4 (66.7)	2 (33.3)	–	–	–	–
	Internal organ or other disability	24 (0.6)	15 (62.5)	9 (37.5)	–	–	–	–
Severity of disability	Severe	135 (3.4)	67 (49.6)	68 (50.4)	*X*^2^ = 1.35 (0.2445)	135 (100.0)	–	–
	Mild	316 (8.0)	138 (43.7)	178 (56.3)		316 (100.0)	–	
Underlying diseases	Stroke	371 (9.4)	134 (36.1)	237 (63.9)	*X*^2^ = 17.13 (<0.0001)	84 (22.6)	287 (77.4)	*X*^2^ = 50.75 (<0.0001)
	Parkinson	28 (0.7)	8 (28.6)	20 (71.4)	*X*^2^ = 0.03 (0.8543)	4 (14.3)	24 (85.7)	*X*^2^ = 0.23 (0.6347)
	Osteoporosis	713 (18.1)	60 (8.4)	653 (91.6)	*X*^2^ = 152.98 (<0.0001)	84 (11.8)	629 (88.2)	*X*^2^ = 0.10 (0.7504)
Charlson comorbidity index^3^	0	2522 (64.0)	610 (24.2)	1912 (75.8)	*X*^2^ = 46.06 (<0.0001)	262 (10.4)	2260 (89.6)	*X*^2^ = 17.82 (0.0001)
	1–2	1293 (32.8)	395 (30.5)	898 (69.5)		161 (12.5)	1132 (87.5)	
	3+	128 (3.2)	61 (47.7)	67 (52.3)		28 (21.9)	100 (78.1)	
Follow-uptime (day), mean (SD)		1939.0 (941.9)	1587.3 (952.6)	1969.3 (936.3)	*t* = −3.32 (0.0009)	1770.4 (932.9)	1960.8 (941.0)	*t* = 4.05 (<0.0001)

^1^ External physical disability includes limb disability, brain disability, visual disability, and auditory impairment; mental disability includes mental retardation and mental disease; internal organ and other disability includes renal impairment and others. ^2^ Household income group were defined as three groups using income decile: Low (0–2), Middle (3–7), High (8–10). ^3^ Individual Charlson comorbidity index was determined for each subject using 2002–2003 medical care utilization records among the study populations.^.^

**Table 3:** Hazard ratios of associated factors with hip fracture and subgroup analysis according to gender and disability.

Variable	Category	Total Hazard Ratio (95% CI)	Gender, Hazard Ratio (95% CI)		Disability, Hazard Ratio (95% CI)	
			Men	Women	Disabled	Non-Disabled
Gender	Men	1.00	–	–	1.00	1.00
	Women	1.49 (1.39–1.61)	–	–	1.28 (1.05–1.56)	1.54 (1.42–1.67)
Age	65–69	1.00	1.00	1.00	1.00	1.00
	70–74	1.67 (1.52–1.83)	1.43 (1.22–1.68)	1.81 (1.62–2.02)	1.11 (0.88–1.40)	1.81 (1.64–2.00)
	75–79	3.04 (2.77–3.32)	2.32 (1.96–2.75)	3.41 (3.06–3.81)	1.51 (1.18–1.94)	3.41 (3.09–3.77)
	80–84	4.17 (3.77–4.60)	3.15 (2.59–3.84)	4.68 (4.16–5.27)	1.57 (1.11–2.23)	4.75 (4.27–5.28)
	85+	4.09 (3.62–4.62)	3.91 (3.04–5.02)	4.30 (3.73–4.97)	1.43 (0.82–2.47)	4.63 (4.07–5.26)
Type of health security	Health insurance	1.00	1.00	1.00	1.00	1.00
	Medical aid	0.52 (0.45–0.59)	0.61 (0.46–0.81)	0.50 (0.43–0.58)	0.51 (0.35–0.73)	0.52 (0.45–0.61)
Household income ^1^	High	1.00	1.00	1.00	1.00	1.00
	Low	1.00 (0.92–1.09)	1.17 (0.98–1.39)	0.94 (0.84–1.04)	1.14 (0.87–1.49)	0.98 (0.89–1.07)
	Middle	0.98 (0.91–1.06)	1.08 (0.94–1.24)	0.94 (0.86–1.02)	0.96 (0.77–1.20)	0.98 (0.90–1.06)
Severity of disability	Non-disabled	1.00	1.00	1.00	–	–
	Severe	1.59 (1.33–1.89)	2.02 (1.57–2.61)	1.28 (1.01–1.64)	–	–
	Mild	1.68 (1.49–1.88)	1.78 (1.48–2.13)	1.60 (1.38–1.87)	–	–
Charlson comorbidity index	0	1.00	1.00	1.00	1.00	1.00
	1–2	1.10 (1.02–1.18)	1.10 (0.96–1.27)	1.10 (1.01–1.20)	0.99 (0.78–1.25)	1.11 (1.03–1.20)
	3+	1.24 (1.03–1.49)	1.45 (1.10–1.92)	1.10 (0.85–1.42)	1.00 (0.64–1.55)	1.27 (1.03–1.56)
Underlying diseases	Stroke (ref = none)	1.27 (1.13–1.43)	1.35 (1.11–1.65)	1.24 (1.08–1.44)	1.48 (1.11–1.98)	1.19 (1.04–1.35)
	Parkinson (ref = none)	1.51 (1.04–2.19)	1.42 (0.70–2.85)	1.56 (1.01–2.43)	0.89 (0.33–2.39)	1.75 (1.17–2.62)
	Osteoporosis (ref = none)	1.35 (1.24–1.46)	1.76 (1.36–2.29)	1.30 (1.19–1.42)	1.47 (1.14–1.90)	1.34 (1.22–1.47)

^1^ Household income group were defined as three groups using income decile: Low (0–2), Middle (3–7), High (8–10).
